# The association between alcohol consumption and the risk of hepatocellular carcinoma according to glycemic status in Korea: A nationwide population-based study

**DOI:** 10.1371/journal.pmed.1004244

**Published:** 2023-06-12

**Authors:** Eun Ju Cho, Goh Eun Chung, Jeong-Ju Yoo, Yuri Cho, Dong Wook Shin, Yoon Jun Kim, Jung-Hwan Yoon, Kyungdo Han, Su Jong Yu

**Affiliations:** 1 Department of Internal Medicine and Liver Research Institute, Seoul National University College of Medicine, Seoul, Republic of Korea; 2 Department of Internal Medicine and Healthcare Research Institute, Seoul National University Hospital Healthcare System Gangnam Center, Seoul, Republic of Korea; 3 Department of Internal Medicine, Division of Gastroenterology and Hepatology, Soonchunhyang University Bucheon Hospital, Gyeonggi-do, Republic of Korea; 4 Center for Liver and Pancreatobiliary Cancer, National Cancer Center, Goyang, Republic of Korea; 5 Department of Family Medicine/Supportive Care Center, Samsung Medical Center, Sungkyunkwan University School of Medicine, Seoul, Republic of Korea; 6 Department of Clinical Research Design and Evaluation/Department of Digital Health, Samsung Advanced Institute for Health Science, Seoul, Republic of Korea; 7 Department of Statistics and Actuarial Science, Soongsil University, Seoul, Republic of Korea

## Abstract

**Background:**

Alcohol and diabetes are known risk factors for hepatocellular carcinoma (HCC); however, it is unclear whether the association between alcohol consumption and HCC risk differs by fasting serum glucose level and diabetes. We investigated the dose–response relationship between alcohol consumption and the risk of HCC according to glycemic status.

**Methods and findings:**

This population-based observational cohort study included patients who underwent general health checkups in 2009 using the Korean National Health Insurance Service Database. The primary outcome was HCC incidence, and Cox proportional hazard regression analysis was performed to estimate the relationship between alcohol consumption and HCC risk according to glycemic status. A total of 34,321 patients newly diagnosed with HCC were observed in the median follow-up period of 8.3 years. In the multivariable model, we adjusted for age, sex, smoking, regular exercise, income, hypertension, dyslipidemia, and body mass index. Mild-to-moderate alcohol consumption increased the risk of HCC in all glycemic statuses (normoglycemia: hazard ratio [HR], 1.06; 95% confidence interval [CI], 1.02 to 1.10; prediabetes: HR, 1.19; 95% CI, 1.14 to 1.24; and diabetes: HR, 2.02; 95% CI, 1.93 to 2.11) compared to normoglycemic nondrinking. Heavy alcohol consumption also increased the risk of HCC in all glycemic statuses (normoglycemia: HR, 1.39; 95% CI, 1.32 to 1.46; prediabetes: HR, 1.67; 95% CI, 1.58 to 1.77; and diabetes: HR, 3.29; 95% CI, 3.11 to 3.49) compared to normoglycemic nondrinking. Since alcohol consumption information in this study was based on a self-administered questionnaire, there may be a possibility of underestimation. Although we excluded patients with a history of viral hepatitis using diagnosis codes, we could not obtain information on hepatitis B or hepatitis C serum markers.

**Conclusions:**

Both mild-to-moderate and heavy alcohol consumption was associated with an increased risk of HCC in all glycemic statuses. The increased risk of HCC according to alcohol consumption was the highest in the diabetes group, suggesting that more intensive alcohol abstinence is required for patients with diabetes.

## Abstract 2

In a large population based cohort study including more than 34,000 people in Korea, Eun Ju Cho and Goh Eun Chung investigate the impact of alcohol consumption on the development of hepatocellular carcinoma according to fasting serum glucose level.

## Author summary

**Why was this study done?:**

- Alcohol and diabetes are known risk factors for hepatocellular carcinoma (HCC).
- It is unclear whether the association between alcohol consumption and HCC risk differs by fasting serum glucose level and diabetes.

**What did the researchers do and find?:**

- We analyzed the health checkup data of the Korean population aged 20 years or older in 2009 provided by the Korean National Health Insurance Service.
- Multivariable Cox proportional hazards regression models were used to evaluate the association of alcohol consumption with the risk of HCC, with a median follow-up duration of 8.3 years.
- In this study, the risk of HCC increased linearly with alcohol consumption in a dose-dependent manner according to glycemic status with synergistic effects between prediabetes/diabetes and alcohol.
- Any amount or frequency of alcohol consumption increased the risk of HCC in patients with diabetes or prediabetes status.

**What do these findings mean?:**

- The increased risk of HCC according to alcohol consumption was the highest in the diabetes group, suggesting that more intensive alcohol abstinence is required for patients with diabetes.
- The interpretation of these results is limited due to potential underestimation of alcohol consumption as the information on alcohol consumption obtained in this study was based on a self-administered questionnaire.

## 1. Introduction

As one of the most common cancers, liver cancer was the third leading cause of cancer-related death worldwide in 2020 [1]. Moreover, hepatocellular carcinoma (HCC) is the most common type of liver cancer, accounting for 80% of all cases [1]. The major risk factors for HCC development are liver cirrhosis, hepatitis B or C virus infection, excess body weight, smoking, diabetes, and heavy alcohol intake [2]. As a result of improved vaccination and the development of antiviral agents, HCC attributable to hepatitis virus infection might be predicted to have declined. Thus, other risk factors, such as alcohol consumption and diabetes, have become increasingly important in HCC development.

Several studies have reported a dose–risk relationship between alcohol and the risk of HCC; however, there is heterogeneity regarding the threshold level of alcohol consumption. In addition, some case–control studies have reported no significant increased risk of HCC among mild-to-moderate drinkers (less than 40 g/day of alcohol in men and 20 g/day in women) compared to heavy drinkers [3,4], and chronic alcohol use >80 g/day for more than 10 years increases the risk of HCC by approximately 5-fold [5]. Conversely, some authors reported that excessive alcohol consumption (>3 drinks/day) was associated with increased HCC and liver disease–related mortality [6]. The American Cancer Society advises that alcohol should be limited to 1 drink/day for women and 2 drinks/day for men or even complete avoidance for cancer prevention [7]. Regarding glycemic status, diabetes doubles the risk of chronic liver disease and HCC [8,9]. Prediabetes or elevated serum fasting glucose levels are also associated with an increased risk of HCC [10–13]. In addition, an excessive amount of circulating blood glucose induces insulin resistance and chronic inflammation, which may result in hepatocellular carcinogenesis [14].

Since both excessive alcohol consumption and diabetes are well-established risk factors of HCC, there is a possibility that the alcohol- and hyperglycemia-induced oxidative stress may synergistically lead to hepatocellular carcinogenesis. However, to our knowledge, no study has investigated the interactions between the quantified amount of alcohol consumption and serum glycemic status on the risk of HCC in the general population because most previous studies did not have enough statistical power to evaluate combinations of more than 2 or 3 categories of alcohol intake and glycemic status. Thus, we investigated the dose–response relationship between alcohol consumption and HCC risk according to glycemic status in a nationwide population-based cohort study.

## 2. Methods

### Ethics statement

The study was performed in accordance with the ethical guidelines of the 1975 Declaration of Helsinki and approved by the Institutional Review Board of Soongsil University approved this study (SSU-202007-HR-236-01). The requirement for written informed consent was waived because anonymous and deidentified information was used for analysis.

### Data source and study setting

This retrospective population-based study was based on the data obtained from the National Health Insurance Service (NHIS) database in Korea and is reported per the Strengthening the Reporting of Observational Studies in Epidemiology (STROBE) guidelines (**S1 Checklist**). A single government insurer provides a mandatory universal insurance system covering approximately 97% of the Korean population, while the remaining 3% of the population is covered by the Medical Aid program [15]. The NHIS database contains information about claims and demographics, medical procedures and treatments, and disease diagnoses submitted by healthcare providers for reimbursement. The National Health Screening Program (NHSP) is offered to all insured persons every 2 years. The NHSP includes a self-reported questionnaire on sociodemographic data, anthropometric measurements, lifestyle behavior, and laboratory testing [16]. All the questionnaire information (including data on alcohol consumption and covariates) and anthropometric measurements (including the fast glucose level) were collected in the baseline survey in 2009.

### Study population

**Fig 1:**
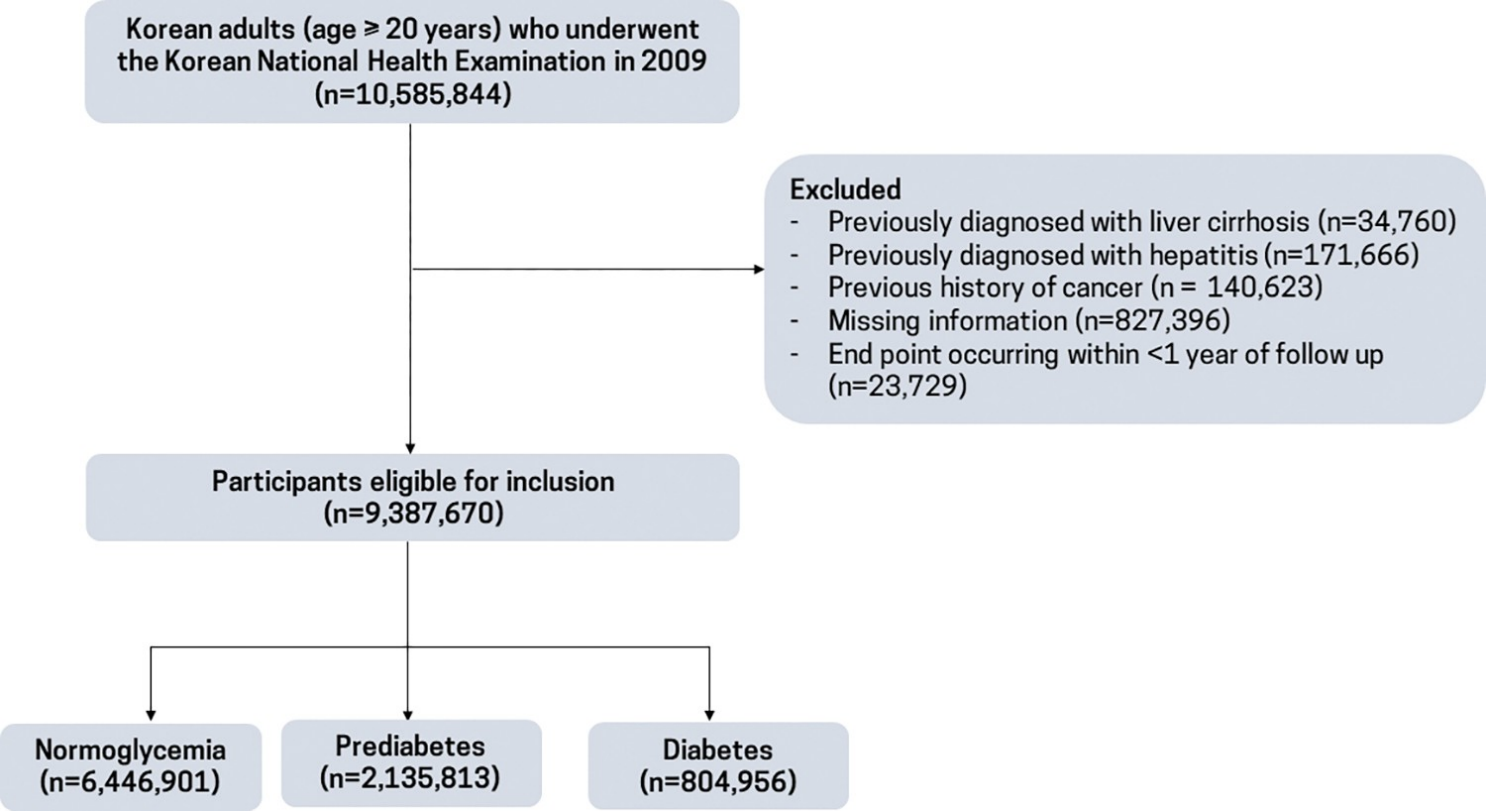
Flow chart of the selection of the study population.

From the NHIS database, we included 10,585,844 Korean adults aged 20 years or older who had participated in the NHSP in 2009. Participants with a previous diagnosis of liver cirrhosis (International Classification of Disease, 10th revision (ICD-10) code, K703 or K746), viral hepatitis (B15 to 19), or cancer (C00 to C97) were excluded. Participants with missing information on sociodemographic data (*n =* 249,517), lifestyle behavior (*n* = 524,121), anthropometric measurement (*n* = 13,493), and laboratory data (*n* = 41,398) were also excluded. In addition, the participants who died or were censored within 1 year of the follow-up period were also excluded. Finally, 9,387,670 individuals were included in the analysis **(Fig 1)**.

### Definition of glycemic status and diabetes

Glycemic status was assessed using serum fasting glucose levels at the time of study enrollment. Prediabetes was defined as a fasting glucose level of 100 to 125 mg/dL [17]. Diabetes was defined as having at least 1 claim per year for a prescription of antidiabetic drugs under the ICD-10 codes E11 to 14 in the insurance claims data or a fasting blood glucose level of 126 mg/dL or higher during the health screening [18]. Finally, the participants were categorized into normoglycemia, prediabetes, and diabetes groups.

### Alcohol consumption

Alcohol consumption was defined based on data on frequency (days per week) and intensity of consumption (number of drinks at each drinking occasion) obtained from a health screening standardized self-reported questionnaire [19]. The frequency of alcohol consumption was categorized as 0, 1 to 2, 3 to 4, and 5 to 7 days/week. A standard unit was defined as a specialized cup for each type of alcohol. Although different drinks can have different alcohol contents, 1 standard unit contains roughly 8 g of ethanol in Korea [20]. Alcohol consumption (g/week) was calculated as the product of frequency and intensity. The participants were divided into 3 groups according to the amount of alcohol intake per week. The 3 groups were as follows: non (0 g/week), mild-to-moderate drinkers (0 to 209 g/week), and heavy drinkers (≥210 g/week) [21].

### Outcome

The primary outcome was HCC incidence, confirmed based on the ICD-10 code: C22.0. The study population was followed up until HCC development, December 31, 2018, or the date of death or emigration, whichever came first.

### Covariates

Smoking status of the participants was classified into nonsmoker, ex-smoker, and current smoker. Regular exercise was defined as when participants exercised at least 3 or more times per week at a high intensity or at least 5 times per week at a moderate intensity. The lowest 25% income proportion was dichotomized into a low-income status. Hypertension was defined with ICD-10 codes (I10 to 13 and I15), taking antihypertensive medications, or systolic blood pressure ≥140 mm Hg or diastolic blood pressure ≥90 mm Hg. Dyslipidemia was defined using ICD-10 code E78, taking lipid-lowering medications, or a total cholesterol level >240 mg/dL [22].

On the day of the health examination, anthropometric measurements, including height, weight, and waist circumference (WC), were performed, and body mass index (BMI) was calculated as weight (kg) divided by height squared (m^2^). Laboratory tests included serum levels of fasting glucose, total cholesterol, triglycerides, high-density lipoprotein cholesterol, and low-density lipoprotein cholesterol. In addition, estimated glomerular filtration rates were calculated from serum creatinine using the Modification of Diet in Renal Disease Study Group [23].

### Statistical analysis

Continuous and categorical variables are expressed as mean ± standard deviation and numbers (%), respectively. For skewed distribution continuous variables, it is expressed as the geometric mean of 95% confidence interval (CI) values. The differences according to glycemic status were tested by analysis of variance for continuous variables and chi-squared tests for categorical variables. The incidence rates of HCC were calculated as the incident cases divided by 1,000 person-years. Cox proportional hazards regression analysis was conducted to estimate hazard ratios (HRs) and 95% CI to estimate the risk of HCC. Multivariable analyses were adjusted for age, sex, smoking history, regular exercise, income, hypertension, dyslipidemia, and BMI. The potential effect modification by covariates, including age, sex, presence of obesity, and smoking status, was evaluated using stratified analysis. The multiplicative interaction between glycemic status and alcohol was tested with the likelihood ratio test. Risk estimates were assessed using age as the time scale in the sensitivity analysis [24].

A negative control outcome shares the same potential sources of bias as the primary outcome but cannot plausibly be related to the conditions of interest [25]. In this observational study, lung cancer was selected a priori for the negative control outcome to detect residual bias from unmeasured confounding. We performed statistical analyses using SAS version 9.4 (SAS Institute, Cary, NC, USA). Statistical significance was defined as a *p*-value < 0.05. The study protocol is presented in S1 Text.

## 3. Results

### Baseline characteristics

**Table 1** shows the baseline characteristics of the study population according to their glycemic status. The prevalence of normoglycemia, prediabetes, and diabetes was 68.7% (6,446,901/9,387,670), 22.8% (2,135,813/9,387,670), and 8.6% (804,956/9,387,670), respectively. The patients in the normoglycemia group were younger than those in the prediabetes and diabetes groups (44.9 versus 49.6 and 57.2 years, respectively) and included more females (49.0% versus 38.3% and 38.9%, respectively). Patients with normoglycemia were more likely to be nonsmokers than other groups. Comorbidities such as hypertension and dyslipidemia were more prevalent in patients with diabetes (*p* < 0.001). In addition, higher BMI and WC values, systolic/diastolic blood pressure, and fasting glucose and triglyceride levels were observed in patients with diabetes compared to those with normoglycemia (*p* < 0.001). The sex-specific clinical features according to the glycemic status showed similar patterns, except for smoking status (**Table A in S1 Appendix**).

**Table 1:** Baseline characteristics of study population according to glycemic status.

	Glycemic status			
	Normoglycemia	Prediabetes	Diabetes	*p*-value
	(*n* = 6,446,901)	(*n* = 2,135,813)	(*n* = 804,956)	
Age, mean ± SD, years	44.9 ± 13.8	49.6 ± 13.2	57.1 ± 12.1	< 0.001
Sex, *n* (%)				< 0.001
Male	3,286,730 (51.0)	1,318,621 (61.7)	491,986 (61.1)	
Female	3,160,171 (49.0)	817,192 (38.3)	312,970 (38.9)	
Smoking, *n* (%)				< 0.001
Non	4,016,086 (62.3)	1,176,949 (55.1)	452,130 (56.2)	
Ex	786,176 (12.2)	363,446 (17.0)	142,135 (17.7)	
Current	1,644,639 (25.5)	595,418 (27.9)	210,691 (26.2)	
Alcohol consumption, *n* (%)				< 0.001
Non	3,352,643 (52.0)	1,005,724 (47.1)	454,239 (56.4)	
Mild-to-moderate	2,607,521 (40.5)	893,128 (41.8)	267,229 (33.2)	
Heavy	486,737 (7.6)	236,961 (11.1)	83,488 (10.4)	
Income low, *n* (%)	1,272,134 (19.7)	404,293 (18.9)	167,151 (20.8)	< 0.001
Regular exercise, *n* (%)	1,095,892 (17.0)	401,429 (18.8)	174,016 (21.6)	< 0.001
Hypertension, *n* (%)	1,243,384 (19.3)	701,517 (32.9)	462,094 (57.4)	< 0.001
Dyslipidemia, *n* (%)	892,065 (13.8)	473,537 (22.2)	329,783 (41.0)	< 0.001
Clinical findings, mean ± SD				
BMI, kg/m^2^	23.3 ± 3.2	24.4 ± 3.2	25.0 ± 3.3	< 0.001
WC, cm	78.8 ± 9.0	82.4 ± 8.7	85.5 ± 8.4	< 0.001
SBP, mm Hg	120.5 ± 14.5	125.8 ± 15.1	129.3 ± 15.9	< 0.001
DBP, mm Hg	75.3 ± 9.9	78.4 ± 10.1	79.2 ± 10.3	< 0.001
Fasting glucose, mg/dL	87.5 ± 7.7	107.8 ± 6.6	147.0 ± 49.7	< 0.001
Total cholesterol, mg/dL	192.7 ± 35.6	202.2 ± 37.7	198.1 ± 42.6	< 0.001
HDL cholesterol, mg/dL	56.8 ± 27.8	55.5 ± 28.0	52.6 ± 29.1	< 0.001
LDL cholesterol, mg/dL	112.6 ± 37.9	117.7 ± 38.7	111.1 ± 43.1	< 0.001
eGFR, mL/min/1.73 m^2^	89.0 ± 48.9	85.1 ± 34.7	83.4 ± 35.8	< 0.001
Triglyceride, mean (95% CI) mg/dL	104.4 (104.4–104.5)	127.7 (127.6–127.8)	150.6 (150.4–150.8)	< 0.001

BMI, body mass index; CI, confidence interval; DBP, diastolic blood pressure; eGFR, estimated glomerular filtration rate; HDL, high-density lipoprotein; LDL, low-density lipoprotein; SBP, systolic blood pressure; WC, waist circumference.

### The risk of incident HCC according to glycemic status and alcohol consumption

Among the total population, 34,321 (0.37%) individuals developed HCC during a median 8.3 (interquartile range, 8.1 to 8.6) years of follow-up. **Table 2** shows the different HR trends by glycemic status and alcohol consumption regarding HCC development. In the age- and sex-adjusted model, the risk of HCC development was increased in mild-to-moderate and heavy drinkers in all the glycemic status groups (normoglycemia: HR, 95% CI = 1.11, 1.07 to 1.14, prediabetes: 1.24, 1.19 to 1.29, and diabetes: 2.07, 1.99 to 2.17 in mild-to-moderate drinkers; normoglycemia: HR, 95% CI = 1.54, 1.46 to 1.62, prediabetes: 1.83, 1.73 to 1.94, and diabetes: 3.55, 3.36 to 3.76 in heavy drinkers) compared to normoglycemic nondrinkers. When further adjusted for smoking, regular exercise, income, hypertension, dyslipidemia, and BMI, these associations remained significant, and the risk of HCC increased linearly with alcohol consumption in a dose-dependent manner in participants with all the glycemic status (normoglycemia: HR, 95% CI = 1.06, 1.02 to 1.10 in mild-to-moderate drinkers and 1.39, 1.32 to 1.46 in heavy drinkers; prediabetes: 1.19, 1.14 to 1.24 in mild-to-moderate drinkers and 1.67, 1.58 to 1.77 in heavy drinkers; and diabetes: 2.02, 1.93 to 2.11 in mild-to-moderate drinkers and 3.29, 3.11 to 3.49 in heavy drinkers). The multiplicative interaction was significant (*p* < 0.001).

**Table 2:** Risk for hepatocellular carcinoma according to glycemic status and alcohol consumption.

Glycemic status	Alcohol consumption	No. of participants	No. of events	Duration	IR per 1,000 PY	HR (95% CI)	
						Age- and sex-adjusted	Multivariable
Normoglycemia	Non	3,352,643	8,992	27,658,134	0.33	1(Ref.)	1(Ref.)
	Mild-to-moderate	2,607,521	6,378	21,573,125	0.30	1.11 (1.07–1.14)	1.06 (1.02–1.10)
	Heavy	486,737	1,944	4,004,437	0.49	1.54 (1.46–1.62)	1.39 (1.32–1.46)
Prediabetes	Non	1,005,724	3,871	8,236,667	0.47	1.04 (1.00–1.08)	1.04 (1.00–1.08)
	Mild-to-moderate	893,128	3,596	7,341,106	0.49	1.24 (1.19–1.29)	1.19 (1.14–1.24)
	Heavy	236,961	1,499	1,936,412	0.77	1.83 (1.73–1.94)	1.67 (1.58–1.77)
Diabetes	Non	454,239	3,807	3,608,812	1.05	1.61 (1.55–1.67)	1.64 (1.58–1.71)
	Mild-to-moderate	267,229	2,802	2,149,864	1.30	2.07 (1.99–2.17)	2.02 (1.93–2.11)
	Heavy	83,488	1,432	665,190	2.15	3.55 (3.36–3.76)	3.29 (3.11–3.49)
*p* for INTm						< 0.001	< 0.001

Multivariable analysis was adjusted for age, sex, smoking, regular exercise, income, hypertension, dyslipidemia, and BMI.

BMI, body mass index; CI, confidence interval; HR, hazard ratio; INTm, multiplicative interaction; IR, incidence rate; PY, person-years.

Sensitivity analyses assessing the risk estimates using age as the time scale showed comparable results (**Table B in S1 Appendix)**. When we performed an analysis stratified by sex, the risk of HCC development was increased in mild-to-moderate and heavy drinkers in all the glycemic status groups in men. In contrast, the dose–response relationships by alcohol were not clear in women with normoglycemia and prediabetes (**Table C in S1 Appendix).**

### The risk of HCC incidence according to glycemic status and alcohol intake pattern: Frequency and amount per occasion

**Table 3** shows the results by alcohol frequency or amount per occasion. In the multivariable analysis, the risk of HCC increased linearly with the frequency of drinking or amount of alcohol consumed per occasion in a dose-dependent manner in patients with prediabetes or diabetes. The risk of HCC, according to the frequency of drinking, was the greatest in patients with diabetes. In the normoglycemia groups, a frequency of drinking 3 or more times per week was associated with an increased risk of HCC. In comparison, a frequency of drinking only 1 to 2 times per week did not show any significant increase in HCC risk. In addition, any amount of alcohol per occasion increased the risk of HCC in patients with diabetes. In contrast, consuming less than 3 glasses per drinking occasion were not associated with an increased HCC risk in those with normoglycemia (**Table 3**).

**Table 3:** Risk for hepatocellular carcinoma according to glycemic status and alcohol frequency or amount per occasion.

Glycemic status	Frequency	No. of participants	No. of events	Duration	IR per 1,000 PY	HR (95% CI)	
						Age- and sex-adjusted	Multivariable
Normoglycemia	0	3,352,643	8,992	27,658,134	0.33	1(Ref.)	1(Ref.)
	1–2	2,352,868	4,650	19,498,914	0.24	1.02 (0.98–1.06)	0.98 (0.94–1.02)
	3–4	548,264	2,092	4,516,972	0.46	1.36 (1.29–1.43)	1.26 (1.20–1.32)
	5–7	193,126	1,580	1,561,677	1.01	1.60 (1.51–1.69)	1.47 (1.40–1.56)
Prediabetes	0	1,005,724	3,871	8,236,668	0.47	1.05 (1.01–1.09)	1.05 (1.01–1.09)
	1–2	751,036	2,388	6,190,882	0.39	1.12 (1.07–1.18)	1.08 (1.03–1.13)
	3–4	264,364	1,465	2,164,732	0.68	1.54 (1.46–1.63)	1.43 (1.36–1.52)
	5–7	114,689	1,242	921,905	1.35	1.90 (1.79–2.02)	1.76 (1.66–1.87)
Diabetes	0	454,239	3,807	3,608,812	1.05	1.63 (1.57–1.69)	1.65 (1.59–1.72)
	1–2	212,310	1,851	1,719,071	1.08	1.92 (1.82–2.02)	1.87 (1.77–1.97)
	3–4	89,012	1,192	712,637	1.67	2.65 (2.49–2.82)	2.51 (2.36–2.67)
	5–7	49,395	1,191	383,347	3.11	3.59 (3.38–3.82)	3.37 (3.17–3.59)
*p* for INTm						< 0.001	< 0.001
Glycemic status	Amount						
Normoglycemia	0	3,352,643	8,992	27,658,134	0.33	1(Ref.)	1(Ref.)
	1–2	486,546	1,247	4,017,460	0.31	1.01 (0.95–1.07)	1.01 (0.95–1.07)
	3–4	697,750	1,957	5,765,131	0.34	1.09 (1.04–1.15)	1.05 (1.00–1.10)
	5–7	1,002,006	3,071	8,274,289	0.37	1.32 (1.27–1.38)	1.23 (1.17–1.28)
	8–14	720,066	1,671	5,963,560	0.28	1.30 (1.23–1.37)	1.18 (1.12–1.25)
	>14	187,890	376	1,557,122	0.24	1.30 (1.17–1.44)	1.15 (1.03–1.28)
Prediabetes	0	1,005,724	3,871	8,236,668	0.47	1.04 (1.00–1.08)	1.04 (1.00–1.08)
	1–2	142,833	630	1,169,612	0.54	1.10 (1.02–1.20)	1.10 (1.02–1.20)
	3–4	232,881	1,154	1,907,658	0.60	1.26 (1.18–1.34)	1.22 (1.15–1.30)
	5–7	401,421	1,996	3,293,513	0.61	1.50 (1.43–1.58)	1.40 (1.33–1.47)
	8–14	280,918	1,089	2,312,783	0.47	1.55 (1.45–1.65)	1.41 (1.32–1.50)
	>14	72,036	226	593,953	0.38	1.45 (1.27–1.66)	1.29 (1.13–1.48)
Diabetes	0	454,239	3,807	3,608,812	1.05	1.60 (1.54–1.66)	1.64 (1.57–1.70)
	1–2	45,165	539	358,433	1.50	1.91 (1.75–2.08)	1.94 (1.78–2.12)
	3–4	72,826	885	581,848	1.52	2.01(1.88–2.16)	1.97 (1.84–2.12)
	5–7	127,907	1,613	1,026,564	1.57	2.55 (2.41–2.69)	2.41 (2.28–2.54)
	8–14	83,003	925	671,697	1.38	2.93 (2.74–3.14)	2.73 (2.54–2.92)
	>14	21,816	272	176,513	1.54	3.73 (3.30–4.21)	3.40 (3.01–3.84)
*p* for INTm						< 0.001	< 0.001

Multivariable analysis was adjusted for age, sex, smoking, regular exercise, income, hypertension, dyslipidemia, and BMI.

BMI, body mass index; CI, confidence interval; HR, hazard ratio; INTm, multiplicative interaction; IR, incidence rate; PY, person-years.

When we performed sensitivity analyses using an age time scale, HCC risk increased linearly with the frequency of drinking or amount of alcohol consumed per occasion in a dose-dependent manner in patients with all glycemic statuses (**Table D in S1 Appendix)**. Increased risk of HCC according to the frequency of drinking or amount of alcohol consumed per occasion among all glycemic statuses remained significant in men (**Tables E and F in S1 Appendix)**.

Next, we employed a negative control outcome. In the study population, 62,443 individuals developed lung cancer. In the multivariable model, there was no significant association between glycemic status and alcohol consumption on the risk of lung cancer (**Table G in S1 Appendix**), supporting no obvious residual bias from unmeasured confounding for the comparison.

### Stratified analysis

We conducted subgroup analyses stratified by age, obesity, and smoking status to confirm the different associations by subgroups. When we performed stratified analyses by age (≥65 years), the presence of obesity (BMI ≥ 25), and smoking status, the increased risk of HCC in heavy drinkers in the diabetes group was generally consistent regardless of baseline characteristics (S1 Fig).

## 4. Discussion

To the best of our knowledge, this is the first study to examine the association between alcohol consumption and HCC risk according to glycemic status. The risk of HCC increased linearly with alcohol consumption in a dose-dependent manner in men in all the glycemic statuses, with synergistic effects between prediabetes/diabetes and alcohol. Additionally, we observed that any amount or frequency of alcohol consumption increased the risk of HCC in patients with diabetes and prediabetes status. In contrast, consuming less than 3 days per week and less than 3 glasses per occasion of drinking were not associated with increased HCC risk in those with normoglycemia. These results suggest an individualized approach in the intervention of alcohol consumption according to the glycemic status for preventing HCC.

The recommendations of several national dietary guidelines regarding low-risk alcohol drinking are based on studies of the risk of alcohol use on all-cause and some cause-specific mortality outcomes [26]. Among patients with diabetes, light-to-moderate alcohol consumption decreases all-cause mortality and cardiovascular disease [27]. In this respect, the American Diabetes Association recommends and limits alcohol consumption to a moderate intake (1 drink per day for females and up to 2 drinks per day for males) [28]. However, there may be different relationships between alcohol use and disease-specific outcomes and may create multiple confounding factors that cannot be easily adjusted in the models analyzing the effect of alcohol on all-cause mortality. Thus, we focused on the influence of alcohol consumption on HCC risk according to glycemic status.

Although excessive alcohol consumption is a well-known risk factor for the development of liver cancer [29], there are inconsistent results regarding the association between mild-to-moderate alcohol consumption and the risk of liver-related mortality or HCC. Among patients with nonalcoholic fatty liver disease (NAFLD), alcohol consumption below 20 g/day was associated with a lesser degree of steatohepatitis and fibrosis [30]. Another study reported that alcohol intake below 20 g/day for women and 40 g/day for men improved insulin resistance and components of metabolic syndrome [31]. However, a recent study reported that alcohol consumption of >96 g/week was associated with advanced fibrosis in NAFLD, and patients with type 2 diabetes had the highest risk of advanced fibrosis [32]. In addition, even mild alcohol consumption (<20 g/day) has been shown to be a risk factor for hepatocarcinogenesis in patients with NAFLD [33].

In this study, mild-to-moderate alcohol consumption and heavy consumption were associated with an increased risk of HCC development, which was greatest in the patients with diabetes, followed by prediabetes and normoglycemia. These results suggest that HCC prevention strategies with respect to alcohol habits should be individualized, considering each patient’s medical condition or comorbidities. For instance, stronger interventions are needed to reduce the risk of HCC in patients with prediabetes or diabetes than in those with normoglycemia.

Regarding frequency and the amount of alcohol consumption, a previous study demonstrated that drinking 3 or more days per week and 5 to 7 units or more per occasion was associated with an increased risk of liver cancers [20]. Similarly, we observed a dose-dependent increment of the weekly drinking frequency and the amount of alcohol consumption per session on the risk of HCC; however, the risk differed according to the patient’s glycemic status, suggesting a threshold dose–risk relationship in normoglycemia and prediabetes but a linear relationship in diabetes.

A linear increase in the risk of HCC according to alcohol consumption in all the glycemic statuses was observed in men; however, this association was not observed in women in this study. These findings may be related to the sex discrepancy in alcohol metabolism. Women have lower alcohol dehydrogenase activity, which increases the systemic ethanol blood level [34]. In addition, since most heavy drinkers were men and HCC incidence in women was low, it is possible that the association of alcohol consumption with HCC risk was less prominent in women than in men.

Several possible mechanisms have been proposed to explain the association between alcohol consumption and HCC according to glycemic status. Acetaldehyde, the metabolic product of ethanol oxidation, is a carcinogenic agent [35]. Moreover, ethanol metabolism is associated with oxidative stress, which promotes the death of hepatocytes and fibrosis development, the precursor condition to cirrhosis [36]. In addition, long-term heavy alcohol consumption could lead to liver cirrhosis, which may progress to HCC [37]. Moreover, hyperglycemia-related conditions, such as chronic oxidative stress and the accumulation of advanced glycosylation end-products, may promote the proliferation and metastatic potential of cancer cells [38,39]. Although the mechanisms of the interaction between alcohol consumption and hyperglycemic status for the development of HCC are unclear, alcohol- and hyperglycemia-related oxidative stress may induce susceptibility to DNA damage and can lead to hepatocellular carcinogenesis. Further studies are warranted to elucidate the pathogenesis regarding the synergistic effect of alcohol consumption and hyperglycemia on HCC development.

This study had several limitations. First, although accurate assessment of alcohol consumption is mandatory, since the alcohol consumption information in this study was based on a self-administered questionnaire, there is a possibility of underestimation of alcohol consumption [40]. Second, although the amount of significant alcohol consumption is not confirmative, the difference by sex was not considered in the definition of the amount of alcohol consumption in this study. Third, since we assessed glycemic and drinking status using the first measurement at baseline, we could not consider the average or change of the status. Fourth, although the aldehyde dehydrogenase 2 gene (ALDH2) polymorphism involves the pathogenesis of HCC [41], we could not obtain this information. In addition, although we excluded patients with a previous diagnosis of viral hepatitis using ICD codes, we could not obtain information on hepatitis B or hepatitis C serum markers. Since the hepatitis B- or C-specific ICD-codes showed moderate sensitivity and moderate positive predictive values [42], further studies based on accurate diagnosis, including serum markers, are needed to generalize the results of this study. Fifth, although we tried to adjust for various HCC-associated covariates, there may be a possible unmeasured remaining confounder, such as healthier behaviors and sociodemographic characteristics associated with high amounts of alcohol. Finally, our data were obtained from an East Asian population of which approximately 30% to 40% are ALDH2 deficient; hence, the results of this study could not be generalized to other ethnic groups. More research is needed to validate our findings.

The risk of HCC from alcohol consumption differed by the baseline glycemic status and was the highest in patients with diabetes. These results suggest an individualized alcohol abstinence strategy by glycemic status for the prevention of HCC in clinical practice. Future studies are needed to elucidate the underlying pathogenesis of the synergistic effect of alcohol consumption and hyperglycemia on HCC development.

In conclusion, mild-to-moderate and heavy alcohol consumption were associated with an increased risk of HCC in all individuals regardless of the glycemic status in men and were associated with an increased risk of HCC in women with diabetes. Patients with diabetes seem to be at the highest risk for HCC, suggesting the need for an individualized alcohol abstinence strategy according to the glycemic status.

## Supporting information

S1 Checklist STROBE statement. (DOCX)

S1 Text Study protocol when applying for use of the data. (DOCX)

S1 Appendix Table A. Baseline characteristics of study population according to glycemic status by sex. Table B. Risk for hepatocellular carcinoma according to glycemic status and alcohol consumption using an age time scale. Table C. Risk for hepatocellular carcinoma according to glycemic status and alcohol consumption stratified by sex. Table D. Risk for hepatocellular carcinoma according to glycemic status and alcohol frequency or amount per occasion using an age time scale. Table E. Risk for hepatocellular carcinoma according to glycemic status and alcohol frequency stratified by sex. Table F. Risk for hepatocellular carcinoma according to glycemic status and alcohol amount per occasion stratified by sex. Table G. Risk for lung cancer according to glycemic status and alcohol consumption.
(DOCX)

S1 Fig Stratified analysis of the risk for hepatocellular carcinoma according to glycemic status and alcohol consumption. BMI, body mass index; DM, diabetes; Normo, normoglycemia.
(JPG)

## Abbreviations

ALDH2: aldehyde dehydrogenase 2 gene
BMI: body mass index
CI: confidence interval
HR: hazard ratio
HCC: hepatocellular carcinoma
ICD-10: International Classification of Disease, 10th revision
NHIS: National Health Insurance Service
NHSP: National Health Screening Program
NAFLD: nonalcoholic fatty liver disease
WC: waist circumference
