# Supplementary material for: The association between alcohol consumption and the risk of hepatocellular carcinoma according to glycemic status in Korea: A nationwide population-based study
Source: PLoS Med. 2023 Jun 12;20(6):e1004244. doi: 10.1371/journal.pmed.1004244 (PMC10259796; doi:10.1371/journal.pmed.1004244)
Supplement: S1 Text — (DOCX) [file pmed.1004244.s002.docx]

**S1 Text. Study protocol when applying for use of the data**

**Study contents**

As one of the most common cancers, liver cancer was the third leading cause of cancer-related death worldwide. Hepatocellular carcinoma (HCC) is the most common type of liver cancer, accounting for 80% of all cases. As a result of improved vaccination and the development of antiviral agents, HCC attributable to hepatitis virus infection might be predicted to have declined. Thus, other risk factors, such as alcohol consumption and diabetes, have become increasingly important in HCC development. However, it is unclear whether the association between alcohol consumption and HCC risk differs by fasting serum glucose level and diabetes. This study aims to investigate the dose-response relationship between alcohol consumption and HCC risk according to glycemic status in Korean adults.

**Study methods**

- Inclusion criteria: Extraction of patients with the corresponding ICD-10 codes and registration codes
- Exclude Criteria: 1) Failure to meet age standards or 2) has already been diagnosed prior to the observation period
- Analysis Method: Using the Cox proportional risk model to investigate and compare the risk of developing HCC by alcohol consumption according to glycemic status

- Subgroup analysis is carried out according to age, sex, obesity and smoking status.

**Expectation effectiveness**- Big Data analysis enable a systematic understanding of modifiable risk factors to prevent HCC.

- Can be used as a basis for drawing social attention and further research and presenting various policies to prevent HCC.
