# Supplementary material for: The association between alcohol consumption and the risk of hepatocellular carcinoma according to glycemic status in Korea: A nationwide population-based study
Source: PLoS Med. 2023 Jun 12;20(6):e1004244. doi: 10.1371/journal.pmed.1004244 (PMC10259796; doi:10.1371/journal.pmed.1004244)
Supplement: S1 Appendix — Table B. Risk for hepatocellular carcinoma according to glycemic status and alcohol consumption using an age time scale. Table C. Risk for hepatocellular carcinoma according to glycemic status and alcohol consumption stratified by sex. Table D. Risk for hepatocellular carcinoma according to glycemic status and alcohol frequency or amount per occasion using an age time scale. Table E. Risk for hepatocellular carcinoma according to glycemic status and alcohol frequency stratified by sex. Table F. Risk for hepatocellular carcinoma according to glycemic status and alcohol amount per occasion stratified by sex. Table G. Risk for lung cancer according to glycemic status and alcohol consumption. (DOCX) [file pmed.1004244.s003.docx]

**The association between alcohol consumption and the risk of hepatocellular carcinoma according to glycemic status in Korea: A nationwide population-based study**

Contents:

Table A. Baseline characteristics of study population according to glycemic status by sex.

Table B. Risk for hepatocellular carcinoma according to glycemic status and alcohol consumption using an age time scale.

Table C. Risk for hepatocellular carcinoma according to glycemic status and alcohol consumption stratified by sex.

Table D. Risk for hepatocellular carcinoma according to glycemic status and alcohol frequency or amount per occasion using an age time scale.

Table E. Risk for hepatocellular carcinoma according to glycemic status and alcohol frequency stratified by sex.

Table F. Risk for hepatocellular carcinoma according to glycemic status and alcohol amount per occasion stratified by sex.

Table G. Risk for lung cancer according to glycemic status and alcohol consumption.

Table A. Baseline characteristics of study population according to glycemic status by sex.

|  | Glycemic status | | | | | | | |  |
| --- | --- | --- | --- | --- | --- | --- | --- | --- | --- |
| Male | Normoglycemia | | Prediabetes | | Diabetes | | *p* value | |  |
|  | (*n* = 3,286,730) | | (*n* = 1,318,621) | | (*n* = 491,986) | |  | |  |
| Age, mean ± SD, years | 43.5 ± 13.2 | | 47.7 ± 12.8 | | 54.9 ± 11.9 | | < 0.001 | |  |
| Smoking, *n* (%) |  | |  | |  | | < 0.001 | |  |
| Non | 1,016,343 (30.9) | | 397,735 (30.2) | | 153,221 (31.1) | |  | |  |
| Ex | 733,672 (22.3) | | 352,199 (26.7) | | 138,364 (28.1) | |  | |  |
| Current | 1,536,715 (46.8) | | 568,687 (43.1) | | 200,401 (40.7) | |  | |  |
| Alcohol consumption, *n* (%) |  | |  | |  | | < 0.001 | |  |
| Non | 1,05,4463 (32.1) | | 382,310 (29.0) | | 179,759 (36.5) | |  | |  |
| Mild-to-moderate | 1,822,598 (55.5) | | 720,732 (54.7) | | 232,861 (47.3) | |  | |  |
| Heavy | 409,669 (12.5) | | 215,579 (16.4) | | 79,366 (16.1) | |  | |  |
| Income low, *n* (%) | 473,997 (14.4) | | 199,712 (15.25) | | 95,874 (19.5) | | < 0.001 | |  |
| Regular exercise, *n* (%) | 619,425 (18.9) | | 268,160 (20.3) | | 118,396 (24.1) | | < 0.001 | |  |
| Hypertension, *n* (%) | 663,207 (20.28) | | 426,178 (32.3) | | 270,252 (54.9) | | < 0.001 | |  |
| Dyslipidemia, *n* (%) | 416,064 (12.7) | | 252,302 (19.1) | | 175,361 (35.6) | | < 0.001 | |  |
| Clinical findings, mean ± SD |  | |  | |  | |  | |  |
| Body mass index, kg/m^2^ | 23.8 ± 3.0 | | 24.5 ± 3.1 | | 24.9 ± 3.1 | | < 0.001 | |  |
| WC, cm | 82.6 ± 7.7 | | 84.7 ± 7.7 | | 86.9 ± 7.9 | | < 0.001 | |  |
| SBP, mmHg | 123.0 ± 13.5 | | 127.0 ± 14.4 | | 129.6 ±1 5.5 | | < 0.001 | |  |
| DBP, mmHg | 77.2 ± 9.5 | | 79.5 ± 9.9 | | 80.0 ± 10.2 | | < 0.001 | |  |
| Glucose, mg/dL | 87.7 ± 7.8 | | 108.1 ± 6.6 | | 150.3 ± 51.1 | | < 0.001 | |  |
| Total cholesterol, mg/dL | 192.4 ± 35.0 | | 199.9 ± 36.7 | | 195.3 ± 41.9 | | < 0.001 | |  |
| HDL cholesterol, mg/dL | 53.4 ± 24.8 | | 53.4 ± 25.6 | | 51.1 ± 27.1 | | < 0.001 | |  |
| LDL cholesterol, mg/dL | 111.8 ± 38.3 | | 114.4 ± 38.5 | | 107.1 ± 43.0 | | < 0.001 | |  |
| eGFR, mL / min / 1.73m^2^ | 89.5 ± 57.1 | | 85.5 ± 38.0 | | 84.9 ± 39.3 | | < 0.001 | |  |
| Triglyceride, mean (95% CI) mg/dL | 121.3 (121.2-121.4) | | 140.6 (140.5-140.8) | | 159.3 (159.0-159.6) | | < 0.001 | |  |
| Female | | Normoglycemia | | Prediabetes | | Diabetes | | *p* value | |
|  |  | (*n* = 3,160,171) | | (*n* = 817,192) | | (*n* = 312,970) | |  | |
| Age, mean ± SD, years | | 46.4 ± 14.2 | | 52.7 ± 13.4 | | 60.8 ± 11.4 | | < 0.001 | |
| Smoking, *n* (%) | |  | |  | |  | | < 0.001 | |
| Non | | 2,999,743 (94.9) | | 779,214 (95.3) | | 298,909 (95.5) | |  | |
| Ex | | 52,504 (1.7) | | 11,247 (1.4) | | 3,771 (1.2) | |  | |
| Current | | 107,924 (3.4) | | 26,731 (3.3) | | 10,290 (3.3) | |  | |
| Alcohol consumption, *n* (%) | |  | |  | |  | | < 0.001 | |
| Non | | 2,298,180 (72.7) | | 623,414 (76.3) | | 274,480 (87.7) | |  | |
| Mild-to-moderate | | 784,923 (24.8) | | 172,396 (21.1) | | 34,368 (11.0) | |  | |
| Heavy | | 77,068 (2.4) | | 21,382 (2.6) | | 4,122 (1.3) | |  | |
| Income low, *n* (%) | | 798,137 (25.3) | | 204,581 (25.0) | | 71,277 (22.8) | | < 0.001 | |
| Regular exercise, *n* (%) | | 476,467 (15.1) | | 133,269 (16.3) | | 55,620 (17.8) | | < 0.001 | |
| Hypertension, *n* (%) | | 580,177 (18.4) | | 275,339 (33.7) | | 191,842 (61.3) | | < 0.001 | |
| Dyslipidemia, *n* (%) | | 476,001 (15.1) | | 221,235 (27.1) | | 154,422 (49.3) | | < 0.001 | |
| Clinical findings, mean ± SD | |  | |  | |  | |  | |
| Body mass index, kg/m^2^ | | 22.8 ± 3.2 | | 24.1 ± 3.4 | | 25.2 ± 3.5 | | < 0.001 | |
| WC, cm | | 74.9 ± 8.5 | | 78.6 ± 8.8 | | 83.3 ± 8.8 | | < 0.001 | |
| SBP, mmHg | | 117.8 ± 15.0 | | 123.9 ± 16.0 | | 128.9 ± 16.5 | | < 0.001 | |
| DBP, mmHg | | 73.3 ± 9.9 | | 76.5 ± 10.3 | | 78.0 ± 10.2 | | < 0.001 | |
| Glucose, mg/dL | | 87.3 ± 7.6 | | 107.3 ± 6.4 | | 141.9 ± 46.9 | | < 0.001 | |
| Total cholesterol, mg/dL | | 192.9 ± 36.2 | | 205.8 ± 38.9 | | 202.5 ± 43.3 | | < 0.001 | |
| HDL cholesterol, mg/dL | | 60.4 ± 30.2 | | 58.9 ± 31.2 | | 54.8 ± 31.9 | | < 0.001 | |
| LDL cholesterol, mg/dL | | 113.4 ± 37.5 | | 123.0 ± 38.5 | | 117.4 ± 42.6 | | < 0.001 | |
| eGFR, mL / min / 1.73m^2^ | | 88.6 ± 38.4 | | 84.4 ± 28.7 | | 80.9 ± 29.3 | | < 0.001 | |
| Triglyceride, mean (95% CI) mg/dL | | 89.3 (89.3-89.4) | | 109.3 (109.2-109.5) | | 137.9 (137.6-138.1) | | < 0.001 | |

SBP, systolic blood pressure; DBP, diastolic blood pressure; WC, waist circumference; HDL, high-density lipoprotein; LDL, low-density lipoprotein; eGFR, estimated glomerular filtration rate; CI, confidence interval

Table B. Risk for hepatocellular carcinoma according to glycemic status and alcohol consumption using an age time scale.

| Glycemic status | Alcohol consumption | | No. of subjects | No. of events | | Duration | IR per 1000 PY | Hazard ratio (95% confidence interval) | |
| --- | --- | --- | --- | --- | --- | --- | --- | --- | --- |
|  |  |  |  |  |  |  |  | Age- and sex- adjusted | Multivariable |
| Normoglycemia | Non | 3,352,643 | | | 8,992 | 27,658,134 | 0.33 | 1(Ref.) | 1(Ref.) |
|  | Mild-to-moderate | 2,607,521 | | | 6,378 | 21,573,125 | 0.30 | 1.13 (1.09–1.17) | 1.08 (1.05–1.13) |
|  | Heavy | 486,737 | | | 1,944 | 4,004,437 | 0.49 | 1.57 (1.49–1.66) | 1.42 (1.34–1.50) |
| Prediabetes | Non | 1,005,724 | | | 3,871 | 8,236,667 | 0.47 | 1.03 (0.99–1.07) | 1.03 (0.99–1.07) |
|  | Mild-to-moderate | 893,128 | | | 3,596 | 7,341,106 | 0.49 | 1.20 (1.15–1.25) | 1.15 (1.10–1.20) |
|  | Heavy | 236,961 | | | 1,499 | 1,936,412 | 0.77 | 1.77 (1.67–1.88) | 1.61 (1.52–1.71) |
| Diabetes | Non | 454,239 | | | 3,807 | 3,608,812 | 1.05 | 1.59 (1.53–1.66) | 1.63 (1.57–1.70) |
|  | Mild-to-moderate | 267,229 | | | 2,802 | 2,149,864 | 1.30 | 1.95 (1.86–2.04) | 1.90 (1.81–1.99) |
|  | Heavy | 83,488 | | | 1,432 | 665,190 | 2.15 | 3.30 (3.10–3.50) | 3.06 (2.88–3.26) |

Multivariable analysis was adjusted for age, sex, smoking, regular exercise, income, hypertension, dyslipidemia and body mass index

IR, incidence rate; PY, person-years

Table C. Risk for hepatocellular carcinoma according to glycemic status and alcohol consumption stratified by sex.

| Glycemic status | Alcohol consumption | | No. of subjects | No. of events | Duration | IR per 1000 PY | Crude HR  (95% CI) | Adjusted HR  (95% CI) |
| --- | --- | --- | --- | --- | --- | --- | --- | --- |
| Male |  | |  |  |  |  |  |  |
| Normoglycemia | Non | | 1,054,463 | 4,509 | 8,606,584 | 0.52 | 1(Ref.) | 1(Ref.) |
|  | Mild-to-moderate | | 1,822,598 | 5,663 | 15,046,867 | 0.38 | 0.72 (0.69–0.75) | 1.10 (1.06–1.14) |
|  | Heavy | | 409,669 | 1,887 | 3,364,771 | 0.56 | 1.07 (1.02–1.13) | 1.46 (1.38–1.54) |
| Prediabetes | Non | | 382,310 | 2,132 | 3,094,695 | 0.69 | 1.32 (1.25–1.39) | 1.06 (1.00–1.11) |
|  | Mild-to-moderate | | 720,732 | 3,306 | 5,911,765 | 0.56 | 1.07 (1.02–1.12) | 1.23 (1.18–1.29) |
|  | Heavy | | 215,579 | 1,467 | 1,759,314 | 0.83 | 1.59 (1.50–1.69) | 1.75 (1.65–1.86) |
| Diabetes | Non | | 179,759 | 2,247 | 1,396,246 | 1.61 | 3.08 (2.93–3.24) | 1.76 (1.67–1.85) |
|  | Mild-to-moderate | | 232,861 | 2,660 | 1,867,263 | 1.42 | 2.72 (2.60–2.86) | 2.11 (2.01–2.22) |
|  | Heavy | | 79,366 | 1,414 | 631,307 | 2.24 | 4.29 (4.04–4.55) | 3.45 (3.25–3.67) |
| *p* for INTm |  | |  |  |  |  | < 0.001 | < 0.001 |
| Female | |  |  |  |  |  |  |  |
| Normoglycemia | | Non | 2,298,180 | 4,483 | 19,051,550 | 0.24 | 1(Ref.) | 1(Ref.) |
|  | | Mild-to-moderate | 784,923 | 715 | 6,526,258 | 0.11 | 0.47 (0.43–0.51) | 0.97 (0.90–1.06) |
|  | | Heavy | 77,068 | 57 | 639,665 | 0.09 | 0.38 (0.29–0.49) | 0.86 (0.66–1.12) |
| Prediabetes | | Non | 623,414 | 1,739 | 5,141,973 | 0.34 | 1.44 (1.36–1.52) | 1.03 (0.97–1.09) |
|  | | Mild-to-moderate | 172,396 | 290 | 1,429,341 | 0.20 | 0.86 (0.77–0.97) | 1.14 (1.01–1.28) |
|  | | Heavy | 21,382 | 32 | 177,098 | 0.18 | 0.77 (0.54–1.09) | 1.06 (0.75–1.50) |
| Diabetes | | Non | 274,480 | 1,560 | 2,212,565 | 0.71 | 2.99 (2.83–3.17) | 1.50 (1.41–1.60) |
|  | | Mild-to-moderate | 34,368 | 142 | 282,601 | 0.50 | 2.14 (1.81–2.52) | 1.68 (1.42–1.99) |
|  | | Heavy | 4,122 | 18 | 33,883 | 0.53 | 2.26 (1.42–3.59) | 2.05 (1.29–3.27) |
| *p* for INTm | |  |  |  |  |  | < 0.001 | < 0.001 |

HR, hazard ratio; CI, confidence interval; IR, incidence rate

Multivariable analysis was adjusted for age, smoking, regular exercise, income, hypertension, dyslipidemia and body mass index

IR, incidence rate; PY, person-years; INTm, multiplicative interaction

Table D. Risk for hepatocellular carcinoma according to glycemic status and alcohol frequency or amount per occasion using an age time scale.

| Glycemic status | Frequency | No. of subjects | No. of events | Duration | IR per 1000 PY | Hazard ratio (95% confidence interval) | |
| --- | --- | --- | --- | --- | --- | --- | --- |
|  |  |  |  |  |  | Age- and sex- adjusted | Multivariable |
| Normoglycemia | 0 | 3,352,643 | 8,992 | 27,658,134 | 0.33 | 1(Ref.) | 1(Ref.) |
|  | 1-2 | 2,352,868 | 4,650 | 19,498,914 | 0.24 | 1.06 (1.01–1.10) | 1.02 (0.98–1.06) |
|  | 3-4 | 548,264 | 2,092 | 4,516,972 | 0.46 | 1.34 (1.27–1.41) | 1.24 (1.18–1.31) |
|  | 5-7 | 193,126 | 1,580 | 1,561,677 | 1.01 | 1.61 (1.52–1.70) | 1.48 (1.39–1.56) |
| Prediabetes | 0 | 1,005,724 | 3,871 | 8,236,668 | 0.47 | 1.03 (0.99–1.07) | 1.03 (0.99–1.08) |
|  | 1-2 | 751,036 | 2,388 | 6,190,882 | 0.39 | 1.09 (1.04–1.14) | 1.05 (1.01–1.10) |
|  | 3-4 | 264,364 | 1,465 | 2,164,732 | 0.68 | 1.47 (1.38–1.56) | 1.37 (1.29–1.46) |
|  | 5-7 | 114,689 | 1,242 | 921,905 | 1.35 | 1.85 (1.74–1.98) | 1.72 (1.61–1.83) |
| Diabetes | 0 | 454,239 | 3,807 | 3,608,812 | 1.05 | 1.61 (1.54–1.67) | 1.64 (1.57–1.71) |
|  | 1-2 | 212,310 | 1,851 | 1,719,071 | 1.08 | 1.79 (1.69–1.89) | 1.75 (1.65–1.84) |
|  | 3-4 | 89,012 | 1,192 | 712,637 | 1.67 | 2.46 (2.30–2.63) | 2.33 (2.18–2.49) |
|  | 5-7 | 49,395 | 1,191 | 383,347 | 3.11 | 3.44 (3.22–3.67) | 3.23 (3.03–3.45) |
| Glycemic status | amount |  |  |  |  |  |  |
| Normoglycemia | 0 | 3,352,643 | 8,992 | 27,658,134 | 0.33 | 1(Ref.) | 1(Ref.) |
|  | 1-2 | 486,546 | 1,247 | 4,017,460 | 0.31 | 1.03 (0.96–1.09) | 1.03 (0.96–1.09) |
|  | 3-4 | 697,750 | 1,957 | 5,765,131 | 0.34 | 1.09 (1.03–1.14) | 1.04 (0.99–1.10) |
|  | 5-7 | 1,002,006 | 3,071 | 8,274,289 | 0.37 | 1.32 (1.26–1.38) | 1.22 (1.17–1.28) |
|  | 8-14 | 720,066 | 1,671 | 5,963,560 | 0.28 | 1.37 (1.30–1.46) | 1.25 (1.18–1.32) |
|  | >14 | 187,890 | 376 | 1,557,122 | 0.24 | 1.49 (1.34–1.66) | 1.33 (1.20–1.49) |
| Prediabetes | 0 | 1,005,724 | 3,871 | 8,236,668 | 0.47 | 1.03 (0.99–1.07) | 1.03 (0.99–1.07) |
|  | 1-2 | 142,833 | 630 | 1,169,612 | 0.54 | 1.08 (0.99–1.18) | 1.08 (0.99–1.18) |
|  | 3-4 | 232,881 | 1,154 | 1,907,658 | 0.60 | 1.21 (1.13–1.29) | 1.17 (1.10–1.25) |
|  | 5-7 | 401,421 | 1,996 | 3,293,513 | 0.61 | 1.43 (1.36–1.51) | 1.33 (1.26–1.41) |
|  | 8-14 | 280,918 | 1,089 | 2,312,783 | 0.47 | 1.50 (1.40–1.61) | 1.37 (1.28–1.47) |
|  | >14 | 72,036 | 226 | 593,953 | 0.38 | 1.52 (1.33–1.75) | 1.36 (1.19–1.57) |
| Diabetes | 0 | 454,239 | 3,807 | 3,608,812 | 1.05 | 1.58 (1.52–1.65) | 1.63 (1.56–1.69) |
|  | 1-2 | 45,165 | 539 | 358,433 | 1.50 | 1.86 (1.70–2.04) | 1.90 (1.73–2.09) |
|  | 3-4 | 72,826 | 885 | 581,848 | 1.52 | 1.92 (1.80–2.06) | 1.89 (1.75–2.03) |
|  | 5-7 | 127,907 | 1,613 | 1,026,564 | 1.57 | 2.35 (2.22–2.49) | 2.23 (2.10–2.36) |
|  | 8-14 | 83,003 | 925 | 671,697 | 1.38 | 2.70 (2.51–2.90) | 2.52 (2.34–2.71) |
|  | >14 | 21,816 | 272 | 176,513 | 1.54 | 3.37 (2.96–3.84) | 3.09 (2.71–3.52) |

Multivariable analysis was adjusted for age, sex, smoking, regular exercise, income, hypertension, dyslipidemia and body mass index.

IR, incidence rate

Table E. Risk for hepatocellular carcinoma according to glycemic status and alcohol frequency stratified by sex.

| Glycemic status | | Frequency | No. of subjects | No. of events | Duration | IR | Crude HR  (95% CI) | | Adjusted HR  (95% CI) |
| --- | --- | --- | --- | --- | --- | --- | --- | --- | --- |
| Male |  | |  |  |  |  |  |  | |
| Normoglycemia | 0 | | 1,054,463 | 4,509 | 8606584 | 0.52 | 1(Ref.) | 1(Ref.) | |
|  | 1-2 | | 1,607,824 | 4,016 | 13303486 | 0.30 | 0.56 (0.55–0.60) | 1.00 (0.96–1.05) | |
|  | 3-4 | | 456,954 | 2,009 | 3758677 | 0.53 | 1.02 (0.97–1.08) | 1.31(1.24–1.38) | |
|  | 5-7 | | 167,489 | 1,525 | 1349475 | 1.13 | 2.16 (2.04–2.29) | 1.51(1.43–1.61) | |
| Prediabetes | 0 | | 382,310 | 2,132 | 3094695 | 0.69 | 1.32 (1.25–1.39) | 1.06 (1.01–1.12) | |
|  | 1-2 | | 592,860 | 2,140 | 4878902 | 0.44 | 0.84 (0.80–0.88) | 1.10 (1.05–1.16) | |
|  | 3-4 | | 238,155 | 1,416 | 1947680 | 0.73 | 1.39 (1.31–1.47) | 1.49 (1.40–1.58) | |
|  | 5-7 | | 105,296 | 1,217 | 844495 | 1.44 | 2.75 (2.58–2.93) | 1.82 (1.71–1.94) | |
| Diabetes | 0 | | 179,759 | 2,247 | 1396246 | 1.61 | 3.08 (2.93–3.24) | 1.77 (1.68–1.87) | |
|  | 1-2 | | 181,142 | 1,733 | 1462295 | 1.19 | 2.26 (2.14–2.39) | 1.94 (1.84–2.06) | |
|  | 3-4 | | 84,050 | 1,169 | 672009 | 1.74 | 3.33 (3.12–3.55) | 2.60 (2.44–2.78) | |
|  | 5-7 | | 47,035 | 1,172 | 364265 | 3.22 | 6.15 (5.77–6.56) | 3.49 (3.27–3.72) | |
| *p* for INTm |  | |  |  |  |  | < 0.001 | < 0.001 | |
| Female |  | |  |  |  |  |  |  | |
| Normoglycemia | 0 | | 2,298,180 | 4,483 | 19051550 | 0.23 | 1(Ref.) | 1(Ref.) | |
|  | 1-2 | | 745,044 | 634 | 6195427 | 0.10 | 0.44 (0.40–0.47) | 0.96 (0.88–1.04) | |
|  | 3-4 | | 91,310 | 83 | 758294 | 0.11 | 0.47 (0.38–0.58) | 0.89 (0.72–1.11) | |
|  | 5-7 | | 25,637 | 55 | 212201 | 0.26 | 1.10 (0.85–1.44) | 1.24 (0.95–1.62) | |
| Prediabetes | 0 | | 623,414 | 1,739 | 5141972 | 0.34 | 1.44 (1.36–1.52) | 1.03 (0.97–1.09) | |
|  | 1-2 | | 158,176 | 248 | 1311979 | 0.19 | 0.81 (0.71–0.91) | 1.11 (0.98–1.27) | |
|  | 3-4 | | 26,209 | 49 | 217050 | 0.23 | 0.96 (0.73–1.27) | 1.23 (0.92–1.63) | |
|  | 5-7 | | 9,393 | 25 | 77409 | 0.32 | 1.37 (0.93–2.03) | 1.16 (0.78–1.72) | |
| Diabetes | 0 | | 274,480 | 1,560 | 2212565 | 0.71 | 2.99 (2.83–3.17) | 1.50 (1.41–1.60) | |
|  | 1-2 | | 31,168 | 118 | 256774 | 0.46 | 1.95 (1.63–2.34) | 1.59 (1.33–1.92) | |
|  | 3-4 | | 4,962 | 23 | 40627 | 0.57 | 2.41(1.60–3.63) | 2.00 (1.33–3.01) | |
|  | 5-7 | | 2,360 | 19 | 19081 | 1.00 | 4.27 (2.72–6.68) | 2.56 (1.64–4.02) | |
| *p* for INTm |  | |  |  |  |  | 0.123 | 0.187 | |

HR, hazard ratio; CI, confidence interval; IR, incidence rate per 1,000 person-years

Multivariable analysis was adjusted for age, smoking, regular exercise, income, hypertension, dyslipidemia and body mass index.

INTm, multiplicative interaction

Table F. Risk for hepatocellular carcinoma according to glycemic status and alcohol amount per occasion stratified by sex.

| Glycemic status | Amount/occasion | No. of subjects | No. of events | IR | Crude HR (95% CI) | Adjusted HR (95% CI) | | |  |
| --- | --- | --- | --- | --- | --- | --- | --- | --- | --- |
| Male |  |  |  |  |  |  | | |  |
| Normoglycemia | 0 | 1,054,463 | 4509 | 0.52 | 1(Ref.) | 1(Ref.) | | |  |
|  | 1-2 | 201,993 | 928 | 0.56 | 1.07 (1.00–1.15) | 1.04 (0.97,1.11) | | |  |
|  | 3-4 | 415,861 | 1685 | 0.49 | 0.94 (0.89–0.99) | 1.07 (1.01,1.13) | | |  |
|  | 5-7 | 789,431 | 2932 | 0.45 | 0.86 (0.82–0.90) | 1.28 (1.22,1.35) | | |  |
|  | 8-14 | 647,725 | 1636 | 0.31 | 0.58 (0.55–0.62) | 1.24 (1.17,1.32) | | |  |
|  | >14 | 177,257 | 369 | 0.25 | 0.48 (0.43–0.53) | 1.21 (1.08,1.34) | | |  |
| Prediabetes | 0 | 382,310 | 2132 | 0.69 | 1.32 (1.25–1.39) | 1.05 (1.00,1.11) | | |  |
|  | 1-2 | 78,630 | 494 | 0.77 | 1.48 (1.35–1.62) | 1.11 (1.01,1.22) | | |  |
|  | 3-4 | 168,261 | 1053 | 0.77 | 1.47 (1.37–1.57) | 1.26 (1.18,1.35) | | |  |
|  | 5-7 | 352,904 | 1933 | 0.67 | 1.28 (1.21–1.35) | 1.50 (1.38,1.54) | | |  |
|  | 8-14 | 266,464 | 1069 | 0.49 | 0.93 (0.87–1.00) | 1.47 (1.38,1.58) | | |  |
|  | >14 | 70,052 | 224 | 0.39 | 0.74 (0.65–0.85) | 1.37 (1.19,1.56) | | |  |
| Diabetes | 0 | 179,759 | 2247 | 1.61 | 3.08 (2.93–3.24) | 1.75 (1.66,1.84) | | |  |
|  | 1-2 | 30,422 | 463 | 1.95 | 3.72 (3.38–4.09) | 2.02 (1.83,2.22) | | |  |
|  | 3-4 | 60,456 | 838 | 1.75 | 3.34 (3.10–3.59) | 2.04 (1.90,2.20) | | |  |
|  | 5-7 | 119,470 | 1582 | 1.65 | 3.16 (2.98–3.35) | 2.50 (2.36,2.65) | | |  |
|  | 8-14 | 80,414 | 920 | 1.41 | 2.70 (2.52–2.90) | 2.87 (2.67,3.08) | | |  |
|  | >14 | 21,465 | 271 | 1.56 | 2.98 (2.64–3.37) | 3.59 (3.17,4.06) | | |  |
| *p* for INTm |  |  |  |  | < 0.001 | < 0.001 | | |  |
| Female |  |  |  |  |  | |  |  |  |
| Normoglycemia | 0 | 2,298,180 | 4483 | 0.24 | 1(Ref.) | | 1(Ref.) |  |  |
|  | 1-2 | 284,553 | 319 | 0.13 | 0.57 (0.51–0.64) | | 0.94 (0.84–1.06) |  |  |
|  | 3-4 | 281,889 | 272 | 0.12 | 0.49 (0.44–0.56) | | 1.04 (0.92–1.18) |  |  |
|  | 5-7 | 212,575 | 139 | 0.08 | 0.34 (0.28–0.40) | | 0.91 (0.76–1.08) |  |  |
|  | 8-14 | 72,341 | 35 | 0.06 | 0.25 (0.18–0.35) | | 0.83 (0.59–1.16) |  |  |
|  | >14 | 10,633 | 7 | 0.08 | 0.34 (0.16–0.71) | | 1.17 (0.56–2.45) |  |  |
| Prediabetes | 0 | 623,414 | 1739 | 0.34 | 1.44 (1.36–1.52) | | 1.03 (0.97–1.09) |  |  |
|  | 1-2 | 64,203 | 136 | 0.26 | 1.09 (0.92–1.29) | | 1.15 (0.97–1.36) |  |  |
|  | 3-4 | 64,620 | 101 | 0.19 | 0.80 (0.66–0.98) | | 1.11 (0.91–1.35) |  |  |
|  | 5-7 | 48,517 | 63 | 0.16 | 0.67 (0.52–0.86) | | 1.10 (0.85–1.41) |  |  |
|  | 8-14 | 14,454 | 20 | 0.17 | 0.71 (0.46–1.10) | | 1.33 (0.86–2.07) |  |  |
|  | >14 | 1,984 | 2 | 0.12 | 0.52 (0.13–2.07) | | 0.92 (0.23–3.66) |  |  |
| Diabetes | 0 | 274,480 | 1560 | 0.71 | 2.99 (2.83–3.17) | | 1.50 (1.41–1.60) |  |  |
|  | 1-2 | 14,743 | 76 | 0.63 | 2.68 (2.13–3.36) | | 1.72 (1.37–2.15) |  |  |
|  | 3-4 | 12,370 | 47 | 0.46 | 1.96 (1.47–2.61) | | 1.69 (1.27–2.26) |  |  |
|  | 5-7 | 8,437 | 31 | 0.45 | 1.90 (1.33–2.70) | | 1.95 (1.37–2.78) |  |  |
|  | 8-14 | 2,589 | 5 | 0.23 | 0.99 (0.41–2.39) | | 1.11 (0.46–2.67) |  |  |
|  | >14 | 351 | 1 | 0.34 | 1.46 (0.21–10.33) | | 1.62 (0.23–11.51) |  |  |
| *p* for INTm |  |  |  |  | 0.289 | | 0.430 |  |  |

Multivariable analysis was adjusted for age, sex, smoking, regular exercise, income, hypertension, dyslipidemia and body mass index.

HR, hazard ratio; CI, confidence interval; IR, incidence rate per 1,000 person-years; INTm, multiplicative interaction Table G. Risk for lung cancer according to glycemic status and alcohol consumption.

| Glycemic status | Alcohol consumption | | No. of subjects | No. of events | Duration | IR per 1000 PY | Hazard ratio (95% confidence interval) | |
| --- | --- | --- | --- | --- | --- | --- | --- | --- |
|  |  |  |  |  |  |  | Age- and sex-adjusted | Multivariable |
| Normoglycemia | Non | 3,352,643 | | 20,531 | 27,631,879 | 0.74 | 1 (Ref.) | 1 (Ref.) |
|  | Mild-to-moderate | 2,607,521 | | 11,276 | 21,563,974 | 0.52 | 0.985 (0.962–1.009) | 0.986 (0.963–1.010) |
|  | Heavy | 486,737 | | 3,289 | 4,002,149 | 0.82 | 0.999 (0.962–1.038) | 0.999 (0.961–1.037) |
| Prediabetes | Non | 1,005,724 | | 8,399 | 8,226,842 | 1.02 | 0.985 (0.960–1.011) | 0.991 (0.966–1.017) |
|  | Mild-to-moderate | 893,128 | | 6,139 | 7,336,192 | 0.84 | 0.975 (0.947–1.004) | 0.981 (0.953–1.011) |
|  | Heavy | 236,961 | | 2,158 | 1,935,169 | 1.12 | 0.997 (0.953–1.043) | 1.001 (0.956–1.047) |
| Diabetes | Non | 454,239 | | 5,728 | 3,605,248 | 1.59 | 1.008 (0.979–1.038) | 1.022 (0.992–1.053) |
|  | Mild-to-moderate | 267,229 | | 3,599 | 2,148,750 | 1.67 | 1.008 (0.973–1.046) | 1.022 (0.985–1.061) |
|  | Heavy | 83,488 | | 1,324 | 665,631 | 1.99 | 1.017 (0.962–1.076) | 1.032 (0.975–1.092) |

Multivariable analysis was adjusted for age, smoking, regular exercise, income, hypertension, dyslipidemia and body mass index.

IR, incidence rate; PY, person-years
