# Supplementary figures and images for: The association between alcohol consumption and the risk of hepatocellular carcinoma according to glycemic status in Korea: A nationwide population-based study
Source: PLoS Med. 2023 Jun 12;20(6):e1004244. doi: 10.1371/journal.pmed.1004244 (PMC10259796; doi:10.1371/journal.pmed.1004244)

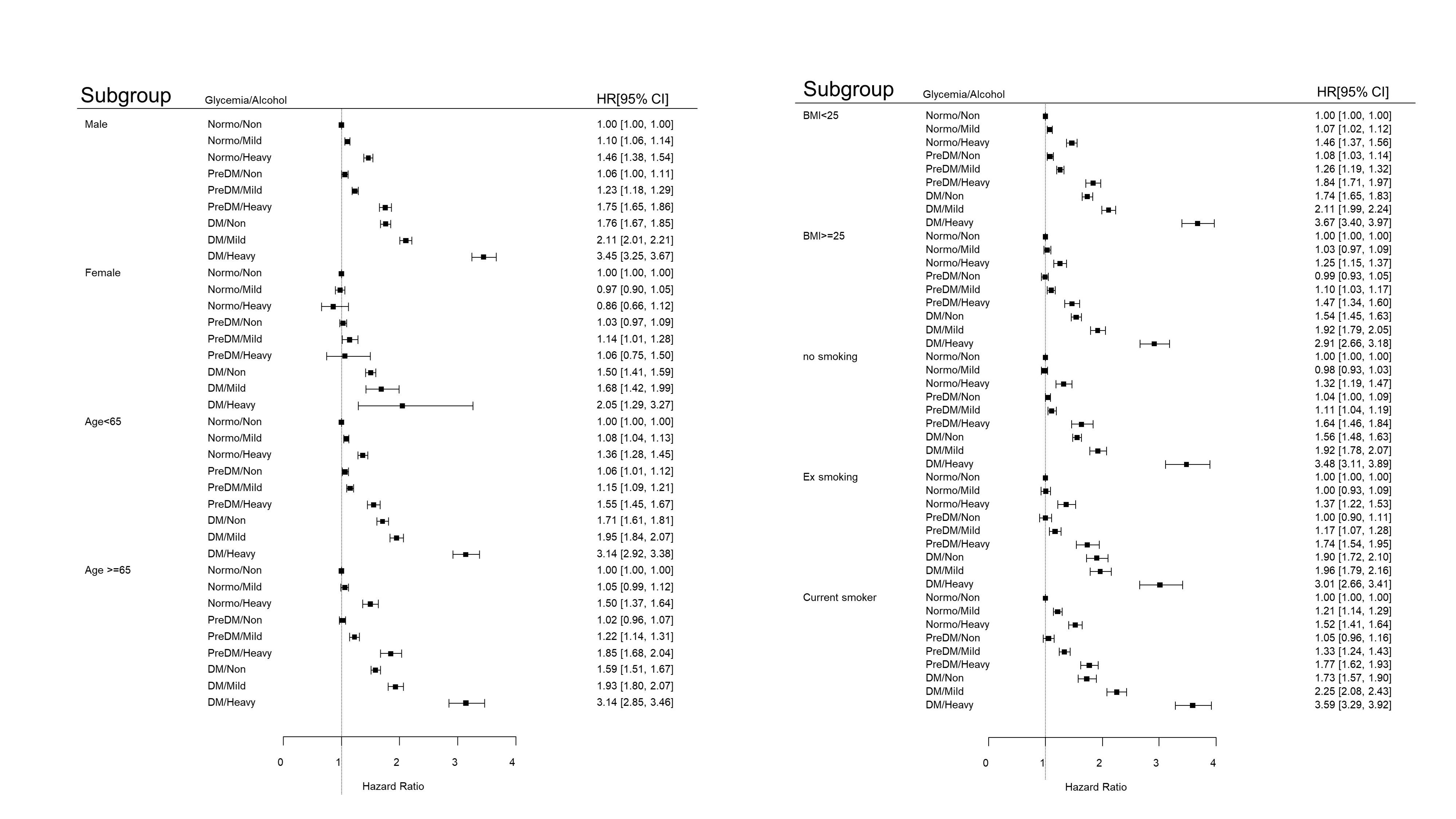

Supplement: S1 Fig — BMI, body mass index; DM, diabetes; Normo, normoglycemia. (JPG) [file pmed.1004244.s004.jpg]
